# Supplementary material for: 3D nanoprinting via spatially controlled assembly and polymerization
Source: Nat Commun. 2022 Apr 11;13:1941. doi: 10.1038/s41467-022-29432-z (PMC9001713; doi:10.1038/s41467-022-29432-z)
Supplement: Supplementary file 1 — Supplementary Information [file 41467_2022_29432_MOESM1_ESM.pdf]

SUPPLEMENTARY INFORMATION

3D Nanoprinting via Spatially Controlled Assembly and Polymerization

Thomas G. Pattison[†], *Shuo Wang*[‡], *Robert D. Miller*[§], *Gang-yu Liu*[‡]*, *Greg G. Qiao*[†]*

[†]Polymer Science Group, Department of Chemical Engineering, The University of Melbourne, Parkville, Victoria 3010, Australia

[‡] Department of Chemistry, University of California, Davis, California 95616, United States

[§] International Business Machines—Almaden Research Center, 650 Harry Road, San Jose, California 95120, United States

Correspondence to:

Gang-yu Liu: gyliu@ucdavis.edu

Greg G Qiao: gregghq@unimelb.edu.au

ORCID

Thomas G. Pattison - https://orcid.org/0000-0002-3029-5896

Greg G. Qiao - http://orcid.org/0000-0003-2771-9675

Gang-yu Liu - http://orcid.org/0000-0003-3689-0685

Table of Contents

Synthesis of p(PEGA-co-HEANB) **3**

Differential scanning calorimetry (DSC) of p(PEGA-co-HEANB) **5**

Formation of 3rd Generation Grubbs catalyst **7**

Surface-initiated ROMP reaction with p(PEGA-co-HEANB) conducted in air **8**

Surface-initiated ROMP reaction with p(PEGA-co-HEANB) when wafer is exposed to air prior to polymer exposure **9**

Robustness of 3D nanoprinting by using a "micropipette" with 4 µm pore **10**

AFM data of polymer printed from 300nm probe with varying probe velocity, used in Figure 3a. **11**

AFM data of polymer printed from 300nm probe, varying printing pressure, used in Figure 3b. **12**

AFM topographic images of lines with varying numbers of passes used to construct them. **13**

References **14**

Synthesis of p(PEGA-co-HEANB)

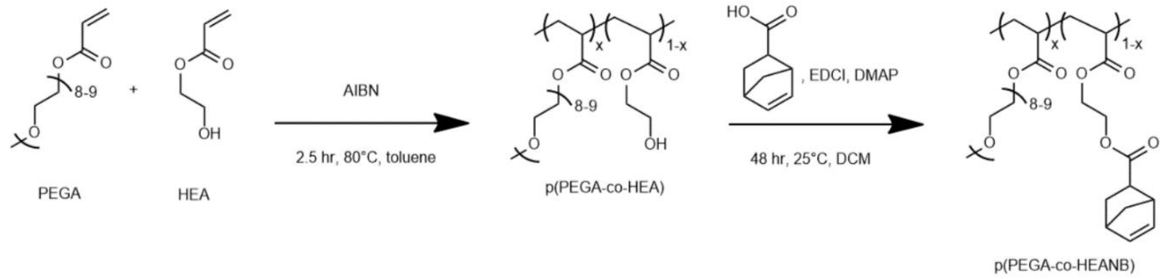

Into a round bottom flask was added hydroxyethyl acrylate (0.283g, 2.44 mmol), polyethylene glycol monomethyl ether acrylate (mw ~480) (1.342g, 2.44 mmol), azobisisobutyronitrile (AIBN) (0.020g, 0.122 mmol) and dry toluene (20 mL). The mixture was stirred and degassed with N_2 for 60 minutes before being submerged in an oil bath set at 80 degrees for 2.5 hours. The round bottom flask was then removed from heat, allowed to cool and the majority of the solvent removed via rotary evaporation. The solution was then dissolved in THF and precipitated in cold pentane. The precipitate was redissolved in THF and precipitated once more into cold pentane. Ether was then added, removed and the precipitate was then placed under vacuum to remove solvent affording P1, a clear viscous liquid. The clear liquid was then combined with *exo*-norbornenecarboxylic acid (0.310g, 2.245 mmol), 4-dimethylaminopyridine (DMAP) (0.047g, 0.345 mmol) and DCM (50 mL), and cooled with an icebath before adding N-(3-dimethylaminopropyl)-N-ethylcarbodiimide hydrochloride (EDCI) (0.397g, 2.073 mmol). The mixture was then stirred for 48 hours before evaporating off the solvent and redissolving in water. The polymer solution was dialyzed for 72 hours against MilliQ water, changing water every two hours initially and 12 hours after the first 3 water changes. The contents of the dialysis tube was then filtered through a 5um, 1um, 0.45um and finally 0.2µm filter before being lyophilized to yield the target polymer p(PEGA-co-HEANB), a transparent, highly viscous liquid. (0.8 g, yield ~60%) 1H NMR (400 MHz, CDCl3): δ 6.13-6.11 (d, 2H), 4.26 (s, 4H), 4.17 (s, 2H), 3.65-3.56 (m, 28H), 3.37 (s, 3H), 3.03 (s, 1H), 2.92 (s, 1H), 2.33 (s, 2H), 2.24 (s, 1H), 1.91 (s, 1H), 1.65 (s, 1H), 1.50-1.48 (s, 2H), 1.36 (q, 1H).

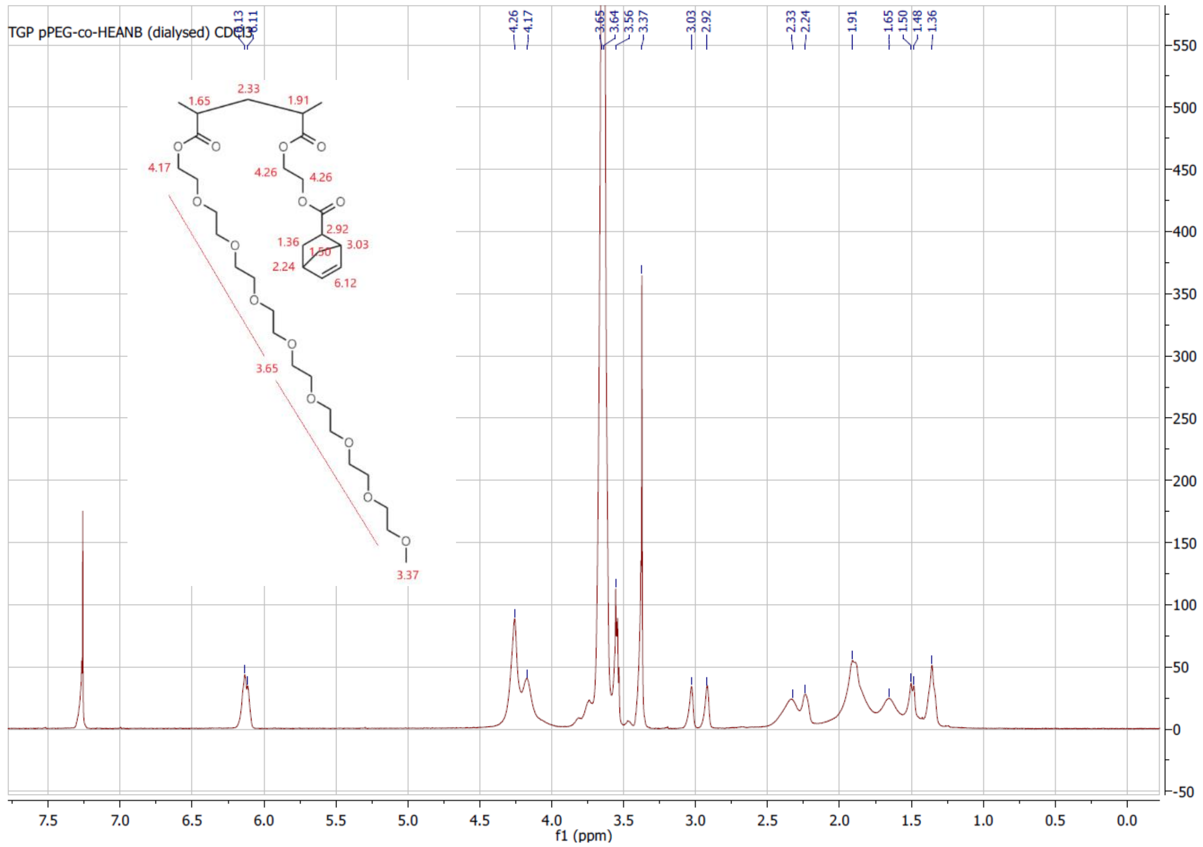

Supplementary Figure 1: H1 NMR of dialyzed p(PEGA-co-HEANB), $CDCl_3$, 298K. The broad peak at 6.2 ppm identify the presence of norbornene olefinic hydrogens, indicating successful post-polymerization functionalization.

Parameter	Value
M_n	24116
M_w	45040
M_p	62314
M_z	65421
PDI	1.86
% side groups with norbornene pendant	50%

Supplementary Table 1: Polymer characteristics of the synthesized p(PEGA-co-HEANB) from GPC analysis including molecular weight, polydispersity. Also included is % norbornene pendants.

Differential scanning calorimetry (DSC) of p(PEGA-co-HEANB)

Limitations of the 3D nano-printing platform used excluded the use of all organic solvents for printing, requiring the printing of material to be performed either with the use of an undissolved liquid material, or a solution in water. The decision to use an undissolved material comes from the limited activity of ROMP in water when used the 3rd generation Grubbs catalyst, as well as the shrinking that can occur when the water mass is lost after printing, resulting in deformation of printed structures.

To do so successfully, the printing material must be a liquid at room temperature. The synthesized p(PEGA-co-HEANB) was a viscous material, and the glass transition temperature is approximately 52°C based on the DSC trace of the first and second heat, indicating that the material's mechanical properties are suitable for printing.

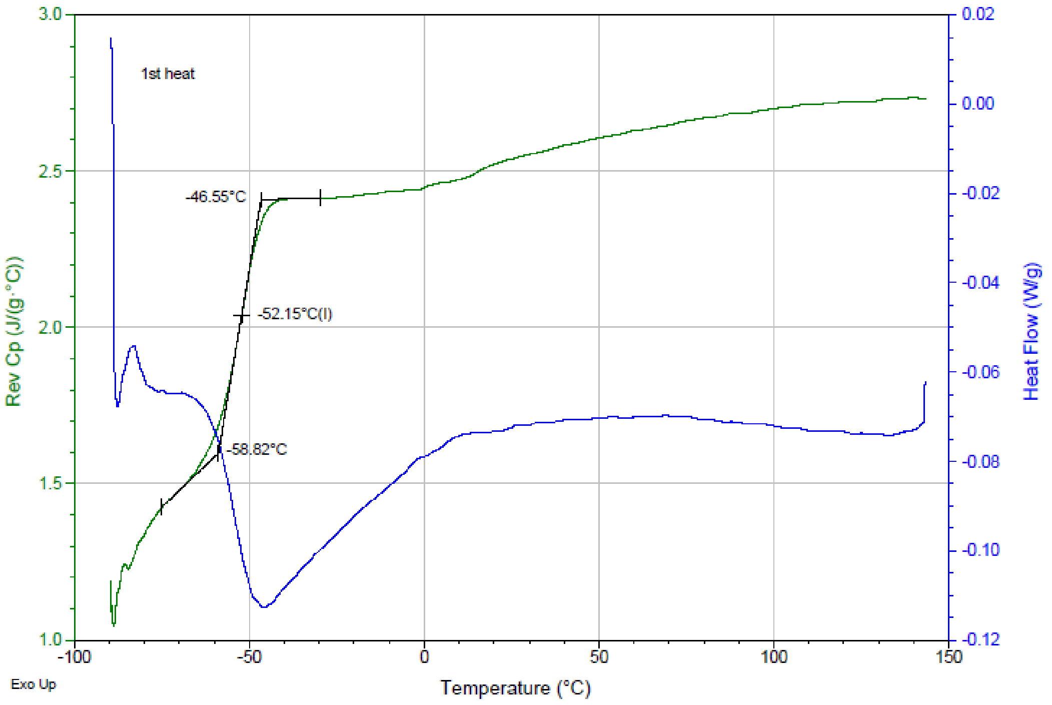

Supplementary Figure 2: DSC of p(PEGA-co-HEANB) (first heat).

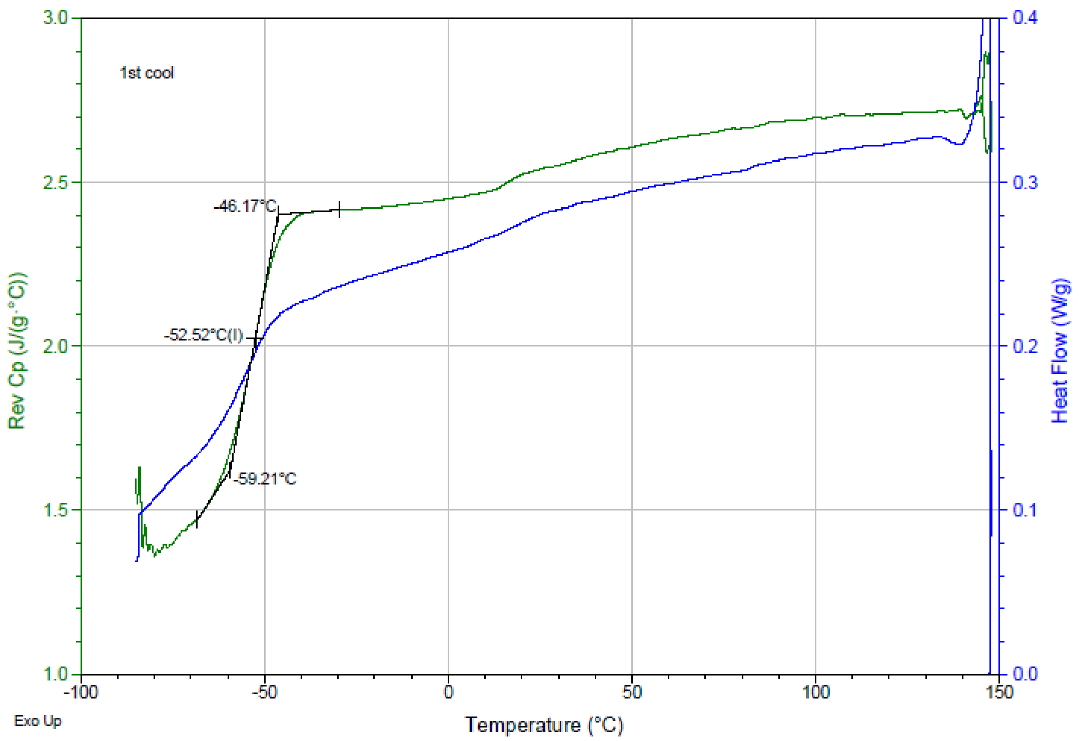

Supplementary Figure 3: DSC of p(PEGA-co-HEANB) (first cool).

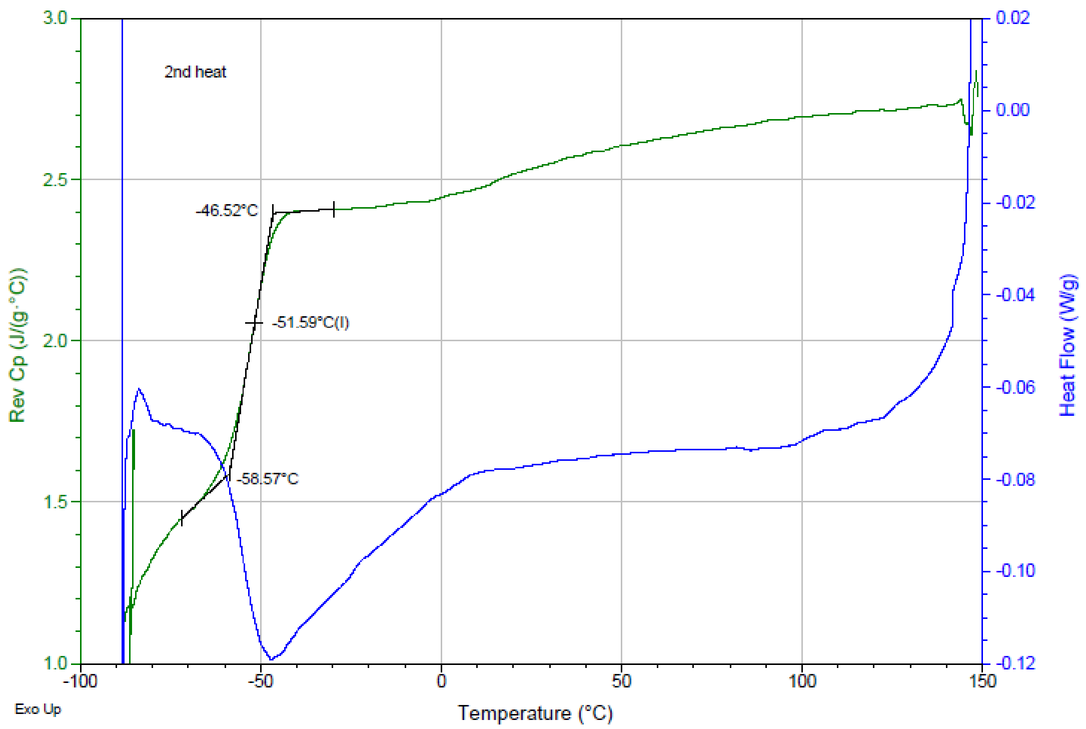

Supplementary Figure 4: DSC of p(PEGA-co-HEANB) (second heat).

Formation of 3rd Generation Grubbs catalyst

The catalyst was synthesized as previously reported[1,2]: Into a vial was added 270mg of 2nd Generation Grubbs Catalyst along with a minimal amount of pyridine (typically ~0.5-1mL, enough to dissolve the majority of the solid). Once dissolved, the now green solution was then filtered through a 0.45µm PFTE filter into 14mL vial before slowly layering pentane onto the top of the pyridine solution so as not to mix the two phases. The phases were stored in the freezer overnight and the material was allowed to precipitate. The green precipitate was separated by vacuum filtration and washed with 5x 15 mL of pentane to remove any remaining pyridine before being transferred to a vial and placed under vacuum where it was transferred to a N_2 filled glove box for storage (160 mg, 70% yield).

The assessment of formation of Grubbs 3rd Generation catalyst is typically done by eye, with a colour change from brick red to bright green confirming the transformation. Further information pertaining to detailed characterisation can be found at the following references[3].

Surface-initiated ROMP reaction with p(PEGA-co-HEANB) conducted in air

To print in a continuous fashion, a rapid method of crosslinking is required – without it, subsequent layers will be deposited onto material that has not yet crosslinked. Surface-initiated ROMP has been shown to crosslink monomers in solution in a rapid fashion, however surface-initiated crosslinking of macrocrosslinkers is more challenging due to increased sterics of the macromolecule[4]. Additionally, it is challenging to conduct ROMP in air[5,6], and due to the nature of the printing setup the printing needed to be conducted in ambient conditions without an inert atmosphere. By using a surface that has been modified with initiator and initiated with catalyst, we tested the efficiency of the crosslinking reaction of p(PEGA-co-HEANB) to ensure that the crosslinking would be rapid enough to print continuously in similar conditions. By exposing the surface to polymer for short time intervals and then rinsing in copious amounts of good solvent (DCM), it was shown that even for as little as five seconds of reaction time significant material was crosslinked and remained on the surface.

**Crosslinker reaction time (seconds)**	**Measured film height (nanometers)**
5	13.8
30	16.1
60	17.3
300	49.8

Supplementary Table 2: Film heights after manually depositing p(PEGA-co-HEANB) onto an initiated silicon wafer vs. duration polymer was kept on wafer before removal with DCM and ethyl vinyl ether, demonstrating the rapid crosslinking of the polymer to the surface.

Surface-initiated ROMP reaction with p(PEGA-co-HEANB) when wafer is exposed to air prior to polymer exposure

The presence of air can negatively impact the ROMP catalyst's activity.[5,6] As wafers were functionalized with catalyst and then transferred to the 3D printing platform which was located in air, there is significant exposure of the initiated catalysed surface to ambient air and moisture prior to and during the printing step. In order to test for the efficacy of the catalyst throughout this process, wafers were initiated with catalyst and either immediately exposed to neat p(PEGA-co-HEANB) for 20 minutes or with a delay for 20 minutes before polymer exposure for 20 minutes. The results in Supplementary Table 2 show the reactivity is similar between wafers that have had prior exposure to air after initiation and those that have not, showing that the catalyst does not exhibit a significant loss in activity even with considerable air exposure. This confirms the reactivity of the system and its suitability for use with the 3D nano-printing platform.

Time in air before reaction (minutes)	Time in air during reaction (minutes)	Measured film height (nanometers)
-	20	129.0
-	20	106.6
20	20	139.5
20	20	104.5

Supplementary Table 3: Film heights after manually depositing p(PEGA-co-HEANB) onto wafers initiated with catalyst which were variably exposed to air prior to polymer exposure.

Robustness of 3D nanoprinting by using a "micropipette" with 4 µm pore

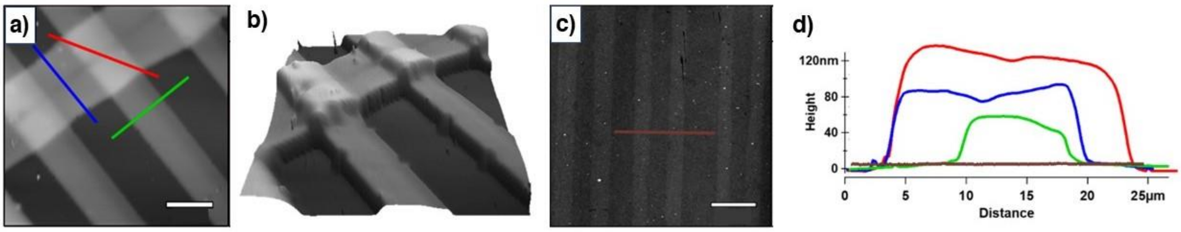

Supplementary Figure 5: Robustness of 3D nanoprinting by using a "micropipette" with 4 µm pore. **a)** 50 µm × 50 µm AFM topographic image of three sets of lines – the 4 lines from top left to bottom right are delivered first. Then a line was delivered by changing the print direction by 90 degrees, resulting in printed lines from middle left to top right of **a)**. The intersection between printed lines can be clearly seen to have an increased height when compared to both individual lines. **b)** 3D display of **a)**, z scale is from 0 to 250 nm. **c)** 50 µm × 50 µm AFM topographic image of printed lines on a surface without catalyst. **d)** Combined cursor profile of **a)** and **c)**. Scale bar in **a)** and **c)**: 10 µm.

AFM data of polymer printed from 300nm probe with varying probe velocity, used in Figure 3a.

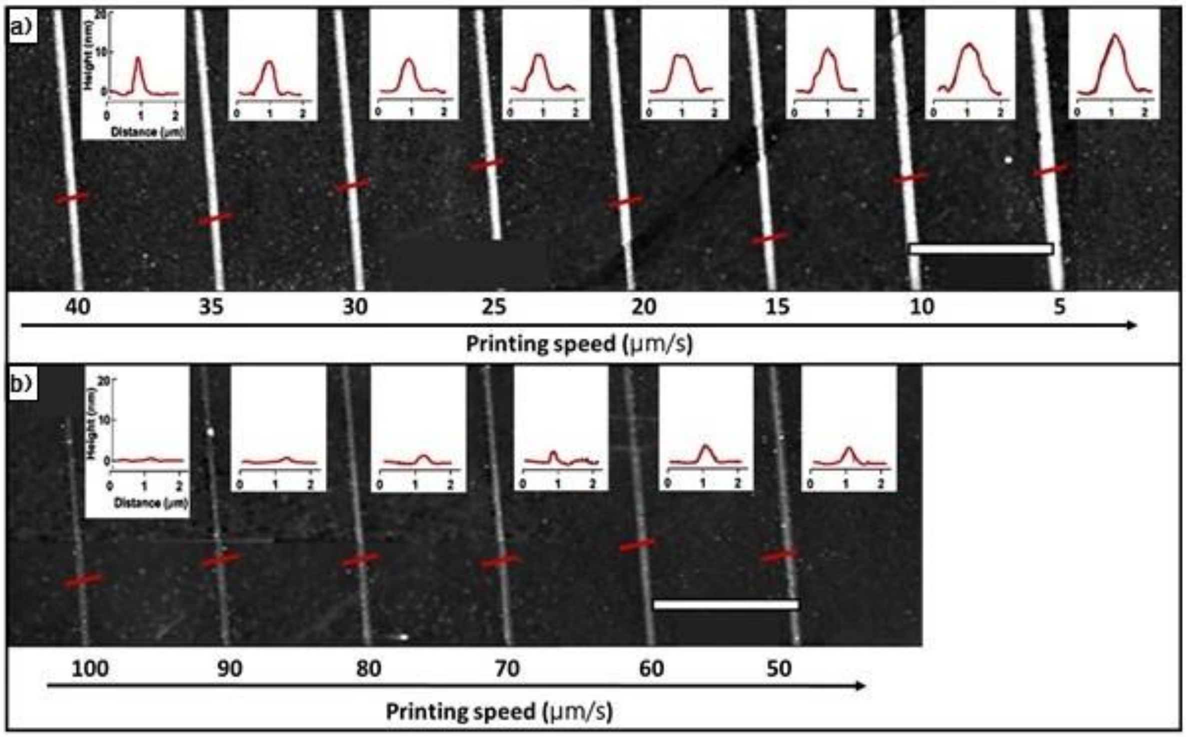

Supplementary Figure 6: AFM topographic images of lines whilst varying the printing speed. The insets are corresponding cursor profiles. **a)** Printing speed varies from 40 µm/s to 5 µm/s. **b)** Printing speed varies from 100 µm/s to 50 µm/s. Scale bars in **a)** and **b)**: 10 µm

AFM data of polymer printed from 300nm probe, varying printing pressure, used in Figure 3b.

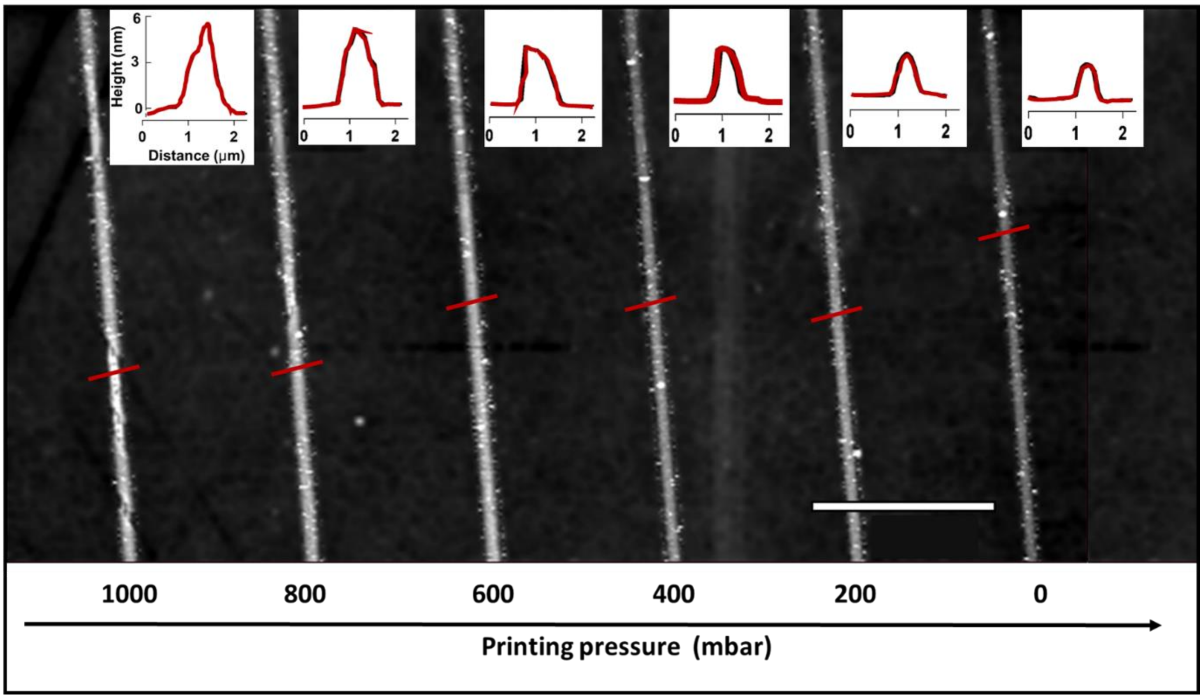

Supplementary Figure 7: AFM topographic images of lines whilst varying the printing pressure. The insets are corresponding cursor profiles. Printing pressure varies from 1000 to 0 mbar. Scale bar: 10 μm.

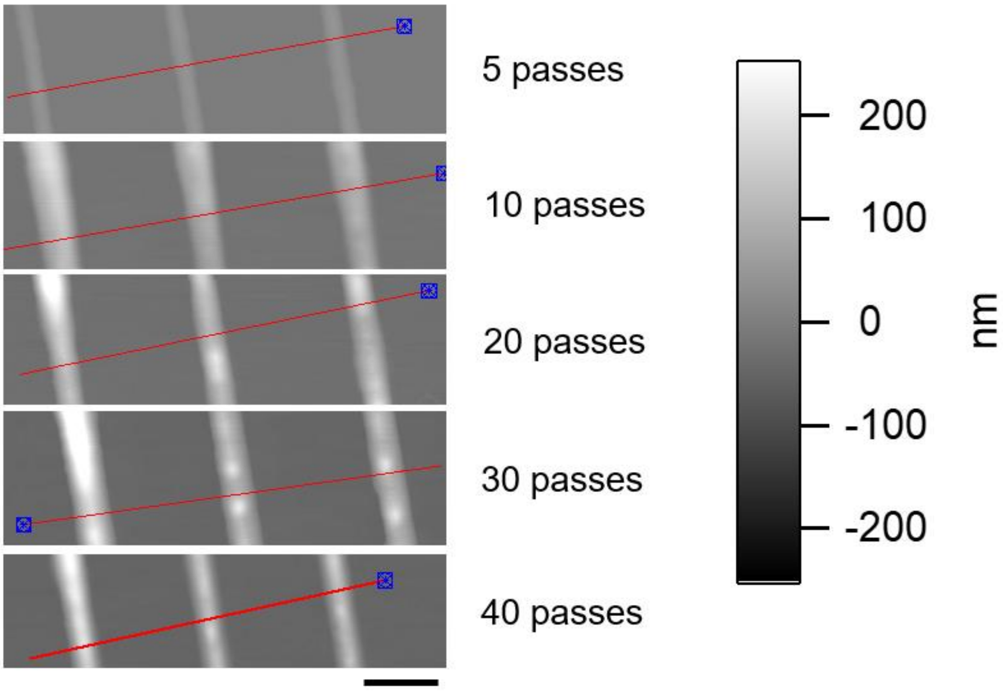

Supplementary Figure 8: AFM topographic images of lines with varying numbers of passes used to construct them. Used to construct Figure 3c. Scale bar: 5 µm.

References

(1) Love, J. A.; Morgan, J. P.; Trnka, T. M.; Grubbs, R. H. A Practical and Highly Active Ruthenium-Based Catalyst That Effects the Cross Metathesis of Acrylonitrile. *Angew. Chem. Int. Ed.* **2002**, *41* (21), 4035–4037. https://doi.org/10.1002/1521-3773(20021104)41:21<4035::AID-ANIE4035>3.0.CO;2-I.

(2) Pattison, T. G.; Spanu, A.; Friz, A. M.; Fu, Q.; Miller, R. D.; Qiao, G. G. Growing Patterned, Cross-Linked Nanoscale Polymer Films from Organic and Inorganic Surfaces Using Ring-Opening Metathesis Polymerization. *ACS Appl. Mater. Interfaces* **2020**, *12* (3), 4041–4051. https://doi.org/10.1021/acsami.9b15852.

(3) Sanford, M. S.; Love, J. A.; Grubbs, R. H. A Versatile Precursor for the Synthesis of New Ruthenium Olefin Metathesis Catalysts. *Organometallics* **2001**, *20* (25), 5314–5318. https://doi.org/10.1021/om010599r.

(4) Nam, E.; Kim, J.; Guntari, S. N.; Seyler, H.; Fu, Q.; Wong, E. H. H.; Blencowe, A.; Jones, D. J.; Caruso, F.; Qiao, G. G. Continuous Assembly of Polymers via Solid Phase Reactions. *Chem. Sci.* **2014**, *5* (9), 3374–3380. https://doi.org/10.1039/C4SC01240B.

(5) Dinger, M. B.; Mol, J. C. Degradation of the First-Generation Grubbs Metathesis Catalyst with Primary Alcohols, Water, and Oxygen. Formation and Catalytic Activity of Ruthenium(II) Monocarbonyl Species. *Organometallics* **2003**, *22* (5), 1089–1095. https://doi.org/10.1021/om0208218.

(6) Dinger, M. B.; Mol, J. C. Degradation of the Second-Generation Grubbs Metathesis Catalyst with Primary Alcohols and Oxygen − Isomerization and Hydrogenation Activities of Monocarbonyl Complexes. *Eur. J. Inorg. Chem.* **2003**, *2003* (15), 2827–2833. https://doi.org/10.1002/ejic.200200702.
